# Mesenchymal stromal cell extracellular vesicles rescue mitochondrial dysfunction and improve barrier integrity in clinically relevant models of ARDS

**DOI:** 10.1183/13993003.02978-2020

**Published:** 2021-07-01

**Authors:** Johnatas Dutra Silva, Yue Su, Carolyn S. Calfee, Kevin L. Delucchi, Daniel Weiss, Danny F. McAuley, Cecilia O'Kane, Anna D. Krasnodembskaya

**Affiliations:** 1 Wellcome-Wolfson Institute for Experimental Medicine, School of Medicine, Dentistry, and Biomedical Sciences, Queen's University Belfast, Belfast, UK; 2 Dept of Medicine, Division of Pulmonary, Critical Care, Allergy and Sleep Medicine, University of California, San Francisco, San Francisco, CA, USA; 3 Dept of Anesthesia, University of California, San Francisco, San Francisco, CA, USA; 4 Cardiovascular Research Institute, University of California, San Francisco, San Francisco, CA, USA; 5 Dept of Psychiatry, University of California, San Francisco, San Francisco, CA, USA; 6 Dept of Medicine, Larner College of Medicine, University of Vermont, Burlington, VT, USA

## Abstract

Alveolar epithelial–capillary barrier disruption is a hallmark of acute respiratory distress syndrome (ARDS). Contribution of mitochondrial dysfunction to the compromised alveolar-capillary barrier in ARDS remains unclear. Mesenchymal stromal cells-derived extracellular vesicles (MSC-EVs) are considered as a cell-free therapy for ARDS. Mitochondrial transfer was shown to be important for the therapeutic effects of MSCs and MSC-EVs. Here we investigated the contribution of mitochondrial dysfunction to the injury of alveolar epithelial and endothelial barriers in ARDS and the ability of MSC-EVs to modulate alveolar–capillary barrier integrity through mitochondrial transfer.

Primary human small airway epithelial and pulmonary microvascular endothelial cells and human precision cut lung slices (PCLSs) were stimulated with endotoxin or plasma samples from patients with ARDS and treated with MSC-EVs, barrier properties and mitochondrial functions were evaluated. Lipopolysaccharide (LPS)-injured mice were treated with MSC-EVs and degree of lung injury and mitochondrial respiration of the lung tissue were assessed.

Inflammatory stimulation resulted in increased permeability coupled with pronounced mitochondrial dysfunction in both types of primary cells and PCLSs. Extracellular vesicles derived from normal MSCs restored barrier integrity and normal levels of oxidative phosphorylation while an extracellular vesicles preparation which did not contain mitochondria was not effective. *In vivo*, presence of mitochondria was critical for extracellular vesicles ability to reduce lung injury and restore mitochondrial respiration in the lung tissue.

In the ARDS environment, MSC-EVs improve alveolar–capillary barrier properties through restoration of mitochondrial functions at least partially *via* mitochondrial transfer.

## Abstract 2

**This study demonstrates that mitochondrial dysfunction is an important mechanism of ARDS pathogenesis. Mitochondrial transfer is crucial for the ability of MSC extracellular vesicles to restore integrity of the alveolar–capillary barrier.** https://bit.ly/2JuqoCY

## Introduction

Acute respiratory distress syndrome (ARDS) is a life-threatening condition characterised by widespread uncontrolled inflammation in the lungs. Damage to the alveolar epithelial–endothelial barrier is a key aspect of ARDS pathophysiology implicated in development of oedema and diffuse alveolar damage [1, 2].

No specific pharmacological treatment is available for patients with ARDS, largely due to the heterogeneity of the underlying pathophysiological mechanisms in different subpopulations of patients [3, 4]. Recently, two biological phenotypes have been identified retrospectively in four randomised clinical trials [5–7] and one observational study [8]. These phenotypes had different clinical characteristics, biomarker profiles, clinical outcomes and, more importantly, they responded differently to interventions (positive end-expiratory pressure, fluid management strategy, low-dose macrolide therapy and simvastatin administration). Although the field is still waiting for the confirmation of these findings in prospective studies, when developing novel therapeutics, it will now be important to consider which ARDS phenotype is present and how it may respond.

Mitochondrial dysfunction and its potential mechanistic role in the pathophysiology of lung diseases, such as chronic obstructive pulmonary disease (COPD), asthma, pulmonary arterial hypertension or idiopathic pulmonary fibrosis, is increasingly recognised [9–11]. Clinical observational data suggest that mitochondrial dysfunction has been associated with higher mortality in critically ill patients with sepsis [12] and survivors of the multiple organ dysfunction syndrome had better mitochondrial function with preservation of ATP and biogenesis markers [13]. However, the role of mitochondrial dysfunction in the pathogenesis of ARDS is not sufficiently studied to date.

Mesenchymal stromal cells (MSCs) are being actively investigated as a potential therapy for ARDS. Accumulating evidence suggests that MSCs act primarily through their secretome, key components of which are extracellular vesicles (EVs) [14, 15]. MSC-EVs are being considered as a cell-free therapy for ARDS [16, 17]. Several publications, including our own studies, have previously reported that MSC-EVs contain mitochondria [18–21]. Since the original finding by Spees *et al.* [22], demonstrating that mitochondrial transfer from MSCs resulted in restoration of aerobic respiration in mitochondria-depleted A549ρ° cells, transfer of MSC-derived mitochondria to pulmonary epithelial cells has been associated with decreased inflammation and injury in the *in vivo* models of acute lung injury [18] and asthma [23]. We have demonstrated that extracellular vesicle-mediated mitochondrial transfer was critical for MSC modulation of primary human macrophages [20]. Furthermore, we have recently found that extracellular vesicle-mediated mitochondrial transfer is required for MSC ability to promote repair of human distal lung epithelial cells [21].

In this study, we explored the contribution of mitochondrial dysfunction to the injury of alveolar epithelial and endothelial barriers in ARDS and the ability of MSC-EVs to modulate alveolar-capillary barrier functions through mitochondrial transfer. We hypothesised that the beneficial effect of MSC-EVs will depend on their ability to alleviate mitochondrial dysfunction and that extracellular vesicles will have differential effects in the microenvironments of hypo- and hyper-inflammatory ARDS phenotypes. Some of the results of these studies were previously reported in the form of an abstract [24].

## Methods

Detailed methods are described in Supplemental Methods.

### Cell culture

Human bone marrow mesenchymal stromal cells (MSCs) were from American Type Culture Collection (ATCC PCS-500-012). These cells met all criteria set by the International Society of Cellular Therapy for the definition of MSCs [25]. Primary human pulmonary microvascular endothelial cells (HPMECs) and human small airway epithelial cells (HSAECs) were from PromoCell (Heidelberg, Germany) and cultured according to manufacturer's instructions.

### Generation of extracellular vesicles from MSC with normal and dysfunctional mitochondria

MSC-EVs were isolated from MSC conditioned medium (CM) by ultracentrifugation according to Zhu *et al.* [26] and characterised according to International Society for Extracellular Vesicles guidelines [27]. To generate MSCs with dysfunctional mitochondria, MSCs were treated with 1 µg·mL^−1^ Rhodamine-6G (Sigma-Aldrich) for 48 h [20] in medium supplemented with 50 ug·mL^−1^ uridine and 2.5 mM sodium pyruvate (Sigma-Aldrich) to support glycolysis, then washed with PBS and incubated for 48 h in serum free medium. extracellular vesicles were isolated from conditioned medium (Rho-EVs) and handled identically to extracellular vesicles isolated from normal MSCs.

### *In vivo* LPS-induced lung injury model

All animal experiments were approved by Animal Welfare Ethical Review Body of Queen's University Belfast, in accordance with UK Animals (Scientific Procedures) Act 1986. C57BL/6 male mice (8–12 weeks old; Envigo RMS UK Ltd, Blackthorn, UK) were used. Mice were anaesthetised by xylazine/ketamine and LPS was instilled intratracheally (2 mg/kg of body weight). 4 h after injury, PBS, extracellular vesicles or Rho-EVs were given *via* tail vein. 24 h after injury, mice were euthanised and bronchoalveolar lavage fluid (BALF) or lungs were taken for analysis or preparation of precision cut lung slices (mPCLSs).

### Human precision-cut lung slices and plasma samples from ARDS patients

Human lungs from organ donors (where the organ had been unsuitable for transplantation, and next of kin had consented for use in research) were used for lung slices cultured *ex vivo*. Ethical approval was given by National Review Ethics Service in association with NHS Blood and Transplant for lungs obtained within the UK (REC 14/LO/0250). Human PCLSs were prepared according to protocol by Uhl *et al*. [28]. Plasma samples used in the study were from patients recruited to HARP-2 study [29]. These samples were previously classified into two phenotypes based on concentrations of plasma inflammatory biomarkers [7]. Only baseline samples obtained prior to intervention were used for experiments. Ten plasma samples representative of each phenotype were pooled and diluted in 1% complete medium to final concentration of 10% before use, plasma from healthy volunteers was used as a control. Ethical approval for use of patient samples for research was granted by the Office for Research Ethics Committees Northern Ireland.

### Statistical analysis

Statistical analysis was performed using Prism 6 software (GraphPad Software, La Jolla, CA, USA). Experiments were done at least in triplicate, the average of three technical replicates was taken as a single data point for each donor, and the points were pooled together for statistical analysis. Pooled data were presented as the mean with standard deviation. For parametric data, a student's test or one-way ANOVA with *post hoc* analysis using Bonferroni's selected comparisons were performed. For nonparametric data, a Kruskal–Wallis test with *post hoc* analysis using Dunn's selected comparisons was used. The statistical significance level was set at p<0.05.

## Results

### MSC-EVs characterisation

Extracellular vesicles were isolated by ultracentrifugation from MSC-conditioned medium generated by MSCs serum starved for 48 h. Viability of MSCs after 48 h of serum starvation was >90%. The dose of extracellular vesicles used in the experiments was based on the final cell count of MSCs, which generated the conditioned medium (10 μL per 1×10^6^ cells). The protein and RNA concentrations of this dose of extracellular vesicles were 98.32±34.6 μg and 31.7±10.7 ng, respectively (n=3).

**FIGURE 1:**
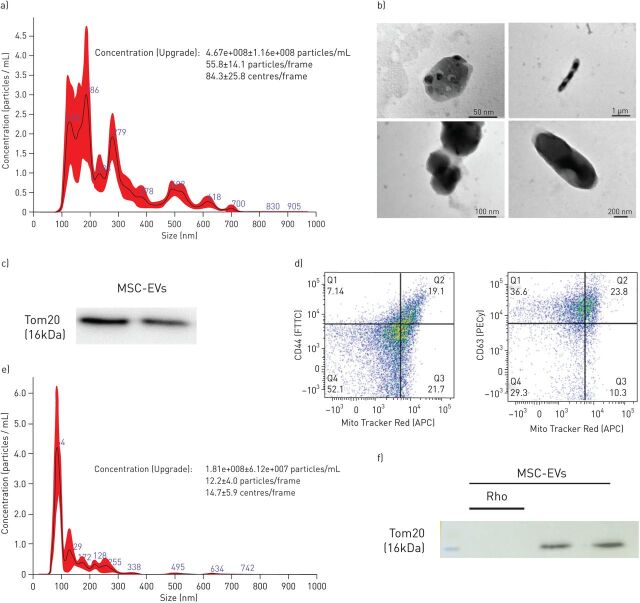
Characterisation of MSC-derived extracellular vesicles (MSC-EVs). a) Characterisation of MSC-EVs using nanoparticle tracking analysis (histogram generated from five independent measurements). b) Representative transmission electron microscopy (TEM) images showing: multivesicular bodies (upper left, scale bar=50 nm), exosomes (bottom left, scale bar=100 nm), mitochondria like structures (upper right, scale bar=1 µm) and mitochondria like structures (bottom right, scale bar 200 nm). The images were taken using TEM microscope (JEOL, JEM 1400Plus, Japan). c) Immunoblot for protein expression levels of TOM20 in MSC-EVs and MSC-EVs-Rho lysates. All lanes were loaded with the same amount of total protein. d) Representative flow cytometry plots of MSC-EVs conjugated with 4 µm beads demonstrating presence of EVs populations double positive for CD44 and MitoTracker Deep Red FM (19.1%) and EV populations double positive for CD63 and MitoTracker Deep Red FM (23.8%). e) Representative histogram of nanoparticle tracking analysis of EVs isolated from Rhodamine-6G pre-treated MSC (histogram generated from five independent measurements). f) Immunoblot for protein expression levels of TOM20 in Rho-EVs lysates. All lanes were loaded with the same amount of total protein as in c.

Nanoparticle tracking analyses (NTA) demonstrated a particle size range of 100–700 nm comprised of two populations of particles with sizes at 100–200 nm corresponding to exosomes, 200–400 nm corresponding to microvesicles, and substantial proportion of larger particles with the sizes at 500, 700 nm and above falling into the size range of mitochondria (figure 1a). Consistently, fine structural analysis by electron microscopy showed presence of exosomes, multi-vesicular bodies and vesicles containing mitochondria (figure 1b). Western blotting of the extracellular vesicle pellet demonstrated pronounced expression of a mitochondrial marker, translocase of outer membrane 20 (Tom20) protein (figure 1c). To further corroborate presence of mitochondria in the extracellular vesicle preparation, extracellular vesicles were isolated from conditioned medium generated from MSCs pre-stained with Mitotracker Deep Red FM, incubated with beads conjugated with anti-CD44 or -CD63Ab and analysed by flow cytometry which demonstrated presence of vesicles double positive for MSC-derived mitochondria and surface markers for MSC-EVs. Quantitatively, CD44^+^Mito^+^ extracellular vesicles constituted 11.3±7.9% of the total extracellular vesicles and CD63^+^Mito^+^ extracellular vesicles constituted 17.6±11.7% of total extracellular vesicles (figure 1d).

In a previous study, we have used Rhodamine-6G to induce specific irreversible inhibition of MSC mitochondrial function [19]. Importantly, this treatment does not affect MSC viability or capacity to secrete paracrine factors. Here we also tested if Rhodamine-6G treatment would affect MSC-EV particle size distribution. NTA demonstrated that the size distribution profile and concentration of Rho-EVs was comparable to the profile of extracellular vesicles isolated from control MSCs except that particles larger than 400 nm, corresponding to the size range of mitochondria, were absent (figure 1e). Consistently, Western blot analysis of Rho-EVs did not detect presence of TOM20 protein (figure 1f). Rho-EVs were used in all the subsequent experiments to investigate the contribution of mitochondria to the overall extracellular vesicle effect.

### Extracellular vesicle mitochondria are readily internalised by HSAECs and HPMECs and restore barrier integrity disrupted by LPS

**FIGURE 2:**
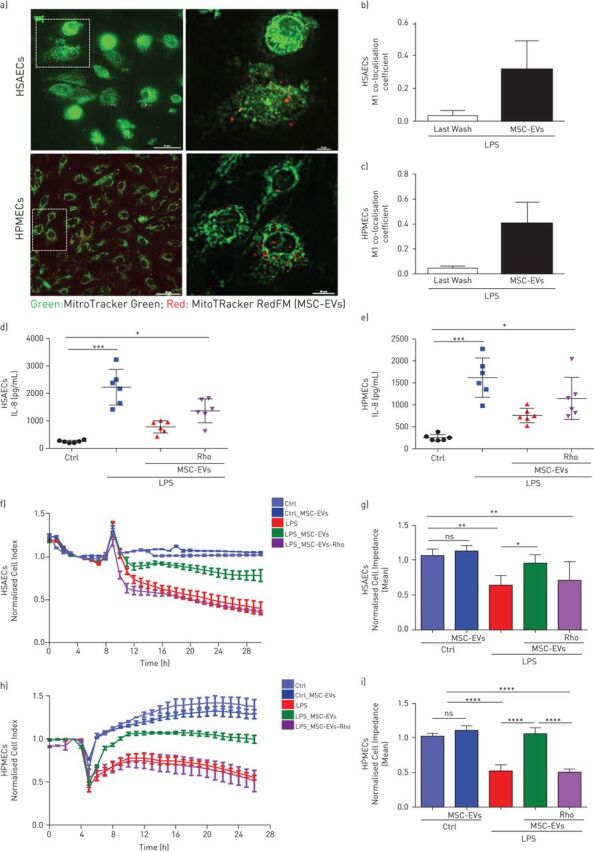
MSC-EVs improve barrier integrity of human primary lung epithelial and endothelial cells through transfer of functional mitochondria. a) Representative live images of MSC-EVs mitochondria internalisation in human small airway epithelial cells (HSAECs) and human pulmonary microvascular endothelial cells (HPMECs) (Scale bars=50 nm (left panel) and 10 nm (right panel)). b and c) M1 coefficient co-localisation in HSAECs (b) and HPMECs (c). The images were taken using a Nikon 6D Eclipse Ti-E inverted microscope with Okolab touch temperature unit and CO_2_ environmental chamber (Nikon Instruments, Japan) (40× dry super plan fluor ELWD objective with 0.6 NA). Data are mean±sd of ten images frames. d and e) Levels of interleukin (IL)-8 secretion by HSAECs (d) and HPMECs (e). f) Representative real-time impedance analysis of HSAECs exposed to lipopolysaccharide (LPS) and treated with MSC-EVs or MSC-EVs-Rho. g) Cell impedance of HSAECs of XCelligence RTCA measurements. Data are mean±sd (n=3). h) Representative real-time impedance analysis of HPMECs exposed to LPS and treated with MSC-EVs or MSC-EVs-Rho. i) Cell impedance of HPMECs of XCelligence RTCA measurements. Data are mean±sd (n=3). ns: not significant; *: p<0.05; **: p<0.01; ***: p<0.001; ****: p<0.0001. Kruskal-Wallis test with *post hoc* Dunn's test (d and e). One-way ANOVA analysis with *post hoc* Bonferroni's test (g and i).

Mitochondrial uptake from MSC-EVs by pulmonary cells was visualised by Nikon Eclipse Ti-E microscopy. EVs were isolated from MSC pre-treated with Mitotracker Deep Red FM and co-cultured with HSAECs or HPMECs that were pre-stained with Mitotracker Green to visualise endogenous mitochondria (figure 2a–c). Three-dimensional reconstruction of multiple z-stacks and deconvolution of images were done using Nikon 6D Eclipse software. At 24 h, there was evidence of robust uptake of MSC-derived mitochondria by both epithelial and endothelial cells (supplementary videos).

As expected, in both HSAECs and HPMECs, LPS stimulation resulted in significant upregulation of interleukin (IL)-8 production. This effect was significantly reversed by both types of extracellular vesicles (figure 2d and e), indicating that mitochondrial transfer is not critical for extracellular vesicle effects on IL-8 production.

LPS stimulation also resulted in significant disruption of both epithelial and endothelial cell barriers as indicated by the alterations in the electrical impedance of the cell monolayer (figure 2f–i). In both cell types, control MSC-EVs were able to restore barrier integrity while Rho-EVs had no effect.

### LPS induces mitochondrial dysfunction in HSAECs and HPMECs which is alleviated by extracellular vesicle mitochondria

**FIGURE 3:**
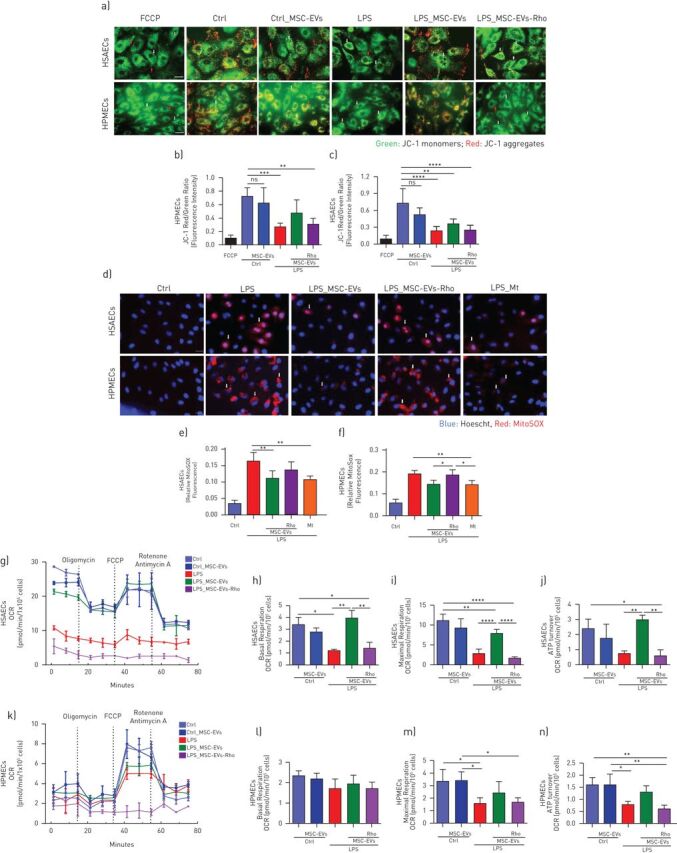
Lipopolysaccharide (LPS) induces mitochondrial dysfunction in human primary lung epithelial and endothelial cells which is alleviated by transfer of functional mitochondria in MSC-EVs. a) Representative live images of JC-1 dye fluorescence as an indicator of mitochondrial membrane potential in human small airway epithelial cells (HSAECs) and human pulmonary microvascular endothelial cells (HPMECs) after exposure to LPS and treatment with MSC-EVs or MSC-EVs-Rho for 24 h. White arrows pointing at the areas of depolarised mitochondrial membranes (JC-1 monomers) and red arrows indicating polarised mitochondrial membranes (JC-1 aggregates). FCCP was used as a control for mitochondria depolarisation. The images were taken using a Nikon 6D Eclipse Ti-E inverted microscope (Scale bar=100 µm). b and c) Quantification of red/green fluorescence intensity ratio in HSAECs (b) and HPMECs (c). Data presented as mean±sd of ten images. d) Representative live images of HSAECs and HPMECs of mitochondrial superoxide production detected with MitoSOX after exposure to LPS and treatment with MSC-EVs or MSC-EVs-Rho for 24 h or pre-treated with MitoTempo (Mt), 4 h prior stimulation. White arrows demonstrating mitochondrial ROS formation in HSAECs and HPMECs. The images were taken using a Nikon 6D Eclipse Ti-E inverted microscope (Scale bar=100 µm). e and f) Quantitative fluorescence intensity of MitoSOX in HSAECs (e) and HPMECs (f). Data presented as mean±sd of ten images. g) Representative Seahorse Mito Stress assay showing oxygen consumption rate (OCR) in HSAECs. h-j) Calculated values for respiratory parameters: basal respiration (h), maximal respiration (i) and ATP production (j), (n=3). k) Representative Seahorse Mito Stress assay showing OCR in HPMECs. l–n) Calculated values for respiratory parameters: basal respiration (l), maximal respiration (m) and ATP production (n), (n=3). Data presented as mean±sd. ns: not significant; *: p<0.05; **: p<0.01; ***: p<0.001; ****: p<0.0001. Kruskal-Wallis test with *post hoc* Dunn's test (c, d, f and g). One-way ANOVA analysis with *post hoc* Bonferroni's test (h–j and l–n).

LPS induced marked decrease in the mitochondrial membrane potential (measured as a ratio between red and green JC-1 dye fluorescence) in both cell types (figure 3a–c). This was accompanied by significant upregulation of the mitochondrial reactive oxygen species (mtROS), (figure 3d–f), collectively demonstrating LPS-induced mitochondrial dysfunction.

EVs were able to partially restore these parameters in both cell types and this effect was dependant on the presence of mitochondria, as Rho-EVs did not have any effect (figure 3a–f).

These findings were further corroborated by assessment of the mitochondrial respiration by measurements of cell oxygen consumption rate, LPS significantly reduced basal and maximum mitochondrial respiration and ATP turnover in both cell types, these effects were partially reversed by control extracellular vesicles but not Rho-EVs (figure 3g–n).

### MSC-EVs reverse LPS-induced changes in mitochondrial quality control mechanisms in HSAECS and HPMECs

**FIGURE 4:**
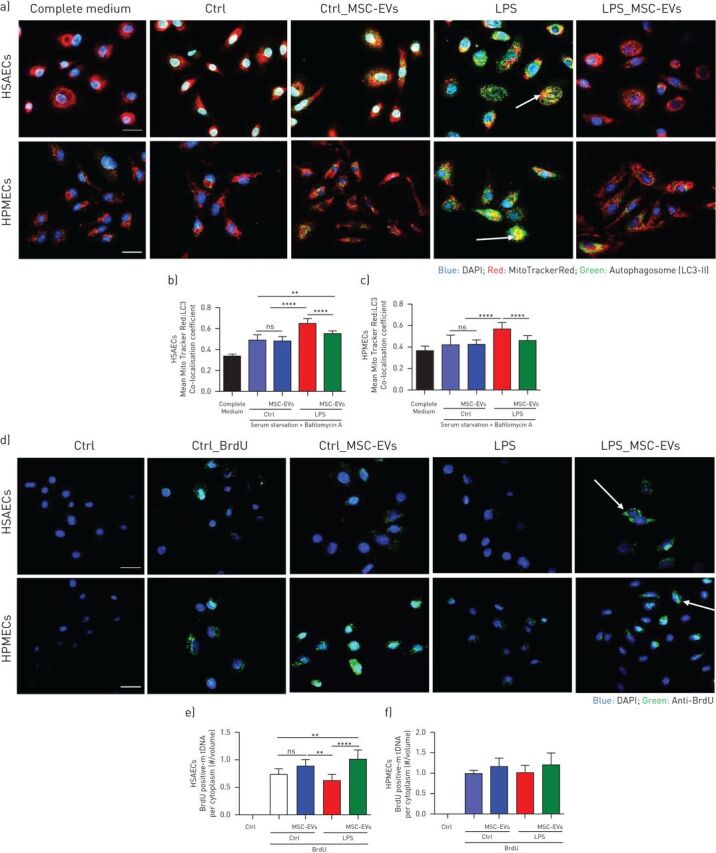
Inflammatory environment perturbs mitochondrial homeostasis in human primary lung epithelial and endothelial cells which is alleviated by MSC-EVs. a) Representative confocal microscopy of human small airway epithelial cells (HSAECs) and human pulmonary microvascular endothelial cells (HPMECs) showing the nucleus (DAPI), mitochondria (red) and LC3-II (green) in different experimental conditions. Arrows indicate co-localisation, indicating incorporation of mitochondria into autophagosome. The images were taken using Leica SP8 confocal microscope with a 40× oil-immersion objective (Scale bar=50 µm). b and c) Mitophagy assessed by co-localisation of mitochondria specific red fluorescence with green autophagosome marker LC3-II in HSAECs (b) and HPMECs (c). Data are mean±sd of ten image frames. d) Representative confocal microscopy showing biogenesis of mitochondria in HSAECs and HPMECs cells. Arrow points at the cytoplasmic accumulation of BrdU-positive mtDNA. The images were taken using Leica SP8 confocal microscope with a 40× oil-immersion objective (Scale bar=50 µm). e and f) Quantification of the ratio of BrdU-positive mtDNA per cytoplasm volume in HSAECs (e) and HPMECs (f). Data are mean±sd of ten image frames. ns: not significant; **: p<0.01; ****: p<0.0001. Kruskal-Wallis test with *post hoc* Dunn's test.

To evaluate effects on mitochondrial homeostasis we assessed levels of mitophagy and mitochondrial biogenesis in epithelial and endothelial cells. Mitophagy was assessed by quantification of co-localisation of mitochondria (Mitotracker Red) to autophagosomes (LC3-II lysosomal marker, green) using confocal microscopy. LPS stimulation induced significant upregulation in the levels of mitophagy in both cell types which were restored to normal levels by MSC-EVs (figure 4a–c).

Mitochondrial biogenesis was assessed by measurements of mtDNA replication levels using BrdU incorporation assay [30]. As opposed to mitophagy, levels of mitochondria biogenesis were significantly inhibited by LPS in HSAECS and treatment with MSC-EVs resulted in restoration of the normal levels of mtDNA replication (figure 4d and e). In HPMECs, although changes in biogenesis levels followed the same trend, effects did not reach statistical significance (figure 4d and f).

### MSC-EVs downregulate LPS-induced inflammatory response and attenuate mitochondrial dysfunction in human PCLSs

**FIGURE 5:**
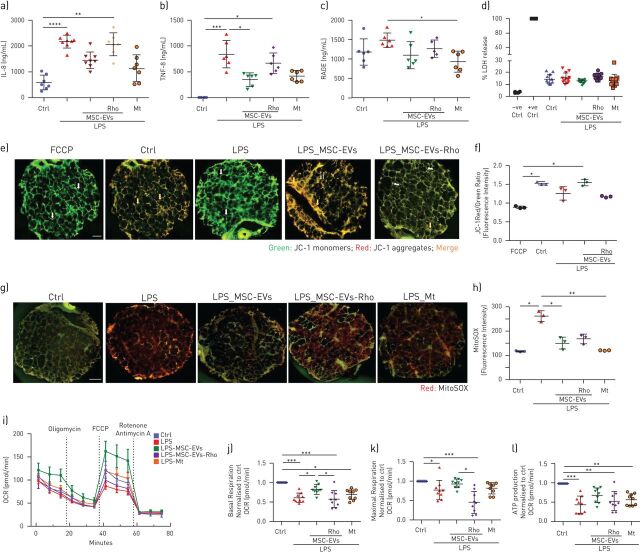
MSC-EVs inhibit inflammatory response and restore mitochondrial function through mitochondrial transfer in human lung tissue in *ex vivo* cultured Human Precision Cut Lung Slices (PCLSs). a) Levels of interleukin(IL)-8 secretion in PCLSs supernatants at 24 h after exposure to LPS (n=6–8, 3 donors). b) Levels of tumour necrosis factor (TNF)-α secretion in PCLSs supernatants at 24 h after exposure to LPS (n=6–8, 3 donors). c) Levels of receptor for advanced glycation end products (RAGE) secretion in PCLSs supernatants at 24 h after exposure to LPS (n=6–8, 3 donors). d) PCLSs viability measured by lactate dehydrogenase (LDH) release (n=8–10, 3 donors). e) Representative live images of JC-1 fluorescence in PCLSs as an indicator of mitochondrial membrane potential. FCCP was used as positive control for complete mitochondrial membrane depolarisation. White arrows point at the areas with low mitochondrial membrane potential (accumulation of JC-1 monomers) and orange arrows point at the areas of normally polarised mitochondrial membranes (accumulation of JC-1 aggregates) in lung tissue. The images were taken using a Nikon 6D Eclipse Ti-E inverted microscope (Scale bar=1 mm). f) Quantification of red to green JC-1 fluorescence ratio in PCLSs was analysed by ImageJ software. Data represented as mean±sd of ten image frames. g) Representative images of mitochondrial superoxide production detected with MitoSOX in PCLSs. The images were taken using a Nikon 6D Eclipse Ti-E inverted microscope (Scale bar=1 mm). h) Quantitative fluorescence intensity was analysed by ImageJ software. Data represented as mean±sd of ten image frames. i) Representative seahorse mito stress test assay showing oxygen consumption rate (OCR) in PCLSs. j–l) Calculated values were normalised to control PCLSs for respiratory parameters: basal respiration (j), maximal respiration (k) and ATP production (l). (n=3 independent experiments, 3 donors). Data represented as mean±sd. ns: not significant; *: p<0.05; **: p<0.01; ***: p<0.001; ****: p<0.0001. Kruskal-Wallis test with *post hoc* Dunn's test.

Next, we investigated the effects of extracellular vesicles on LPS-induced mitochondrial dysfunction in the more physiologically relevant human lung tissue model. Human precision cut lung slices (PCLSs) were exposed to LPS and treated with extracellular vesicles for 24 h. Mitochondrial transfer to PCLSs was visualised by confocal microscopy (supplementary figure S1). LPS stimulation resulted in significant upregulation of IL-8 and tumour necrosis factor (TNF)-α secretion levels in PCLS conditioned medium, which were significantly alleviated by control extracellular vesicles but not Rho-EVs (figure 5a and b). LPS also induced significant upregulation of the receptor for advanced glycation end products (RAGE) levels indicating alveolar epithelial injury [30]. Addition of control but not Rho-EVs restored RAGE levels to the baseline (figure 5c). Importantly, LPS stimulation did not induce cell death (as measured by LDH release) suggesting that observed changes in cytokine secretion were not accountable to passive leakage from necrotic cells (figure 5d). Addition of a specific mtROS inhibitor, MitoTempo, resulted in downregulation of TNF-α, IL-8 and RAGE secretion recapitulating the effect of control extracellular vesicles, suggesting that inhibition of mtROS may be partially responsible for the anti-inflammatory effect of extracellular vesicle mitochondrial transfer (figure 5a–d).

Exposure of PCLSs to LPS for 24 h induced significant depolarisation of the mitochondrial membrane potential and upregulation of mtROS. Both of these endpoints were restored by administration of control extracellular vesicles but not Rho-EVs (figure 5e–h). Consistently, analysis of PCLS oxygen consumption rate demonstrated LPS-induced inhibition of basal and maximal mitochondrial respiration and ATP turnover. Addition of control but not Rho-EVs was able to restore mitochondrial respiration (figure 5i–l). Treatment with MitoTempo did not have a significant effect on restoration of mitochondrial respiration in PCLSs suggesting that inhibition of mtROS alone is not enough to alleviate LPS-induced mitochondrial dysfunction at the lung tissue level.

### MSC-EVs modulate barrier properties and inflammatory response of the HSAECS and HPMECs when stimulated with plasma samples from patients with ARDS; hypo-inflammatory ARDS environment is more responsive to the extracellular vesicle treatment

To mimic human ARDS environment more closely and to investigate extracellular vesicle effects in the different ARDS phenotypes, HSAECs and HPMECs were cultured for 24 h in the presence of 10% plasma samples of patients with ARDS previously classified into hypo- or hyper-inflammatory phenotypes [7].

**FIGURE 6:**
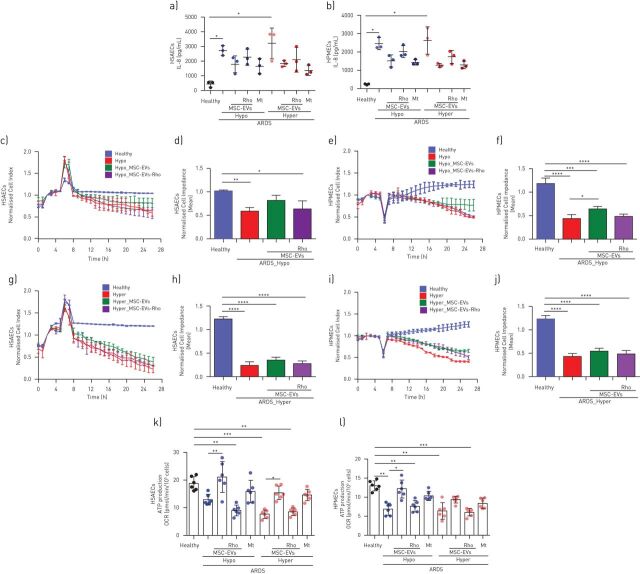
MSC-EVs modulate inflammatory response and partially restore epithelial and endothelial barrier properties *via* mitochondrial transfer in ARDS environment *in vitro*. a, b) Levels of interleukin (IL)-8 secretion by human small airway epithelial cells (HSAECs) (a) and human pulmonary microvascular endothelial cells (HPMECs) (b) at 24 h in both types of acute respiratory distress syndrome (ARDS) plasma (n=3). c) Representative real-time impedance analysis of HSAECs exposed to hypo-inflammatory ARDS plasma. d) Cell impedance analysis of XCelligence RTCA measurements in HSAECs (n=3). e) Representative real-time impedance of HPMECs exposed to hypo-inflammatory ARDS plasma. f) Cell impedance analysis of XCelligence RTCA measurements in HPMECs (n=3). g) Representative real-time impedance of HSAECs exposed to hyper-inflammatory ARDS plasma. h) Cell impedance analysis of XCelligence RTCA measurements in HSAECs (n=3). i) Representative real-time impedance analysis of HPMECs exposed to hyper-inflammatory ARDS plasma samples. j) Cell impedance analysis of XCelligence RTCA measurements in HPMECs (n=3). k and l). Calculated values for mitochondrial ATP production of HSAECs (k) and HPMECs (l) in both ARDS microenvironments (n=3). Data represented as mean±sd. *: p<0.05; **: p<0.01; ***: p<0.001; ****: p<0.0001. Kruskal-Wallis test with *post hoc* Dunn's test.

Exposure to both types of plasma samples elicited comparable increases in IL-8 secretion in both epithelial and endothelial cells. Addition of both control and Rho-EVs comparably reduced ARDS plasma-stimulated increases (figure 6a and b). Addition of MitoTempo resulted in strong inhibition of IL-8 secretion in both cell types and both ARDS phenotypes.

Assessment of the barrier function demonstrated that exposure to each type of ARDS plasma induced comparable disruption of the barrier integrity in HSAECs and HPMECs (figure 6c–j). This was partially restored by extracellular vesicles only in the case of hypo-inflammatory plasma, Rho-EVs had no effect (figure 6c–f).

Exposure to both types of ARDS plasma significantly impaired mitochondrial ATP turnover in HSAECs and HPMECs (figure 6k and l). In both cell types, MSC-EVs were able to significantly attenuate defects in the mitochondrial respiration in the presence of hypo-inflammatory plasma, whereas in hyper-inflammatory plasma extracellular vesicle effect was partial or absent. Rho-EVs did not have any effect on the mitochondrial respiration. Treatment with MitoTempo was not able to significantly improve mitochondrial ATP turnover (figure 6k and l).

### Angiopoietin-2

**FIGURE 7:**
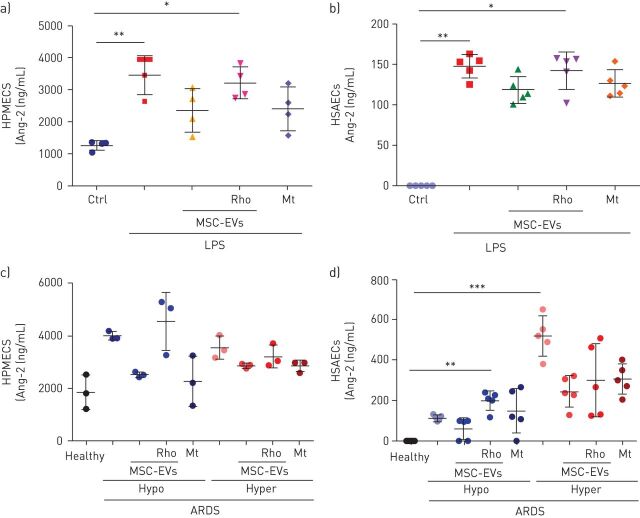
MSC-EVs downregulate levels of angiopoetin (Ang)-2 secretion by human primary lung epithelial and endothelial cells. a) Levels of Ang-2 secretion in human pulmonary microvascular endothelial cells (HPMECs) supernatants at 24 h after exposure to LPS (n=4) and treated with PBS, MSC-EVs, MSC-EVs-Rho or Mitotempo (Mt). b) Levels of Ang-2 secretion in human small airway epithelial cells (HSAECs) supernatants at 24 h after exposure to LPS (n=5) and treated with PBS, MSC-EVs, MSC-EVs-Rho or Mitotempo (Mt). c) Levels of Ang-2 secretion in HPMECs supernatants at 24 h in both types of acute respiratory distress syndrome (ARDS) plasma (n=3) and treated with PBS, MSC-EVs, MSC-EVs-Rho or Mitotempo (Mt). d) Levels of of Ang-2 secretion in HSAECs supernatants at 24 h in both types of ARDS plasma (n=5) and treated with PBS, MSC-EVs, MSC-EVs-Rho or Mitotempo (Mt). Data represented as mean±sd. *: p<0.05; **: p<0.01; ***: p<0.001. Kruskal-Wallis test with *post hoc* Dunn's test.

Angiopoietin-2 (Ang-2) is a well-recognised mediator and biomarker of pulmonary and systemic vascular injury [31–33]. Plasma concentrations of Ang-2 have important predictive value for the development of ARDS and robustly predict poor clinical outcomes in adults and children with ARDS [34, 35]. Importantly, statistically significant decrease in plasma Ang-2 concentrations at 6 h after MSC infusion was reported in the START study (a randomised phase 2a safety trial of MSCs in ARDS) [36]. Therefore, we were interested in the effects of extracellular vesicles on Ang-2 secretion levels by endothelial and epithelial cells in our models. LPS stimulation resulted in robust upregulation of Ang-2 secretion by both HPMECs and SAECs (of note, SAECs had numerically almost 10-fold lower levels of Ang-2 secretion compared with HPMECs), this was partially restored by control extracellular vesicles and MitoTempo but not Rho-EVs (figure 7a and b). Consistently, stimulation with ARDS plasma also elicited an increase in Ang-2 secretion by both cell types. In HPMECs, control extracellular vesicles but not Rho-EVs were able to partially reduce its levels in the presence of hypo-inflammatory plasma whereas no effect was detected in the presence of hyper-inflammatory plasma (figure 7c). In SAECs, control extracellular vesicles but not Rho-EVs were able to partially restore Ang-2 levels in the presence of hypo-inflammatory plasma whereas addition of both control and Rho-EVs comparably reduced Ang-2 concentrations in the presence of hyper-inflammatory plasma (figure 7d).

### MSC-EVs attenuate lung injury and restore lung tissue mitochondrial respiration in the mouse model of LPS-induced lung injury

**FIGURE 8:**
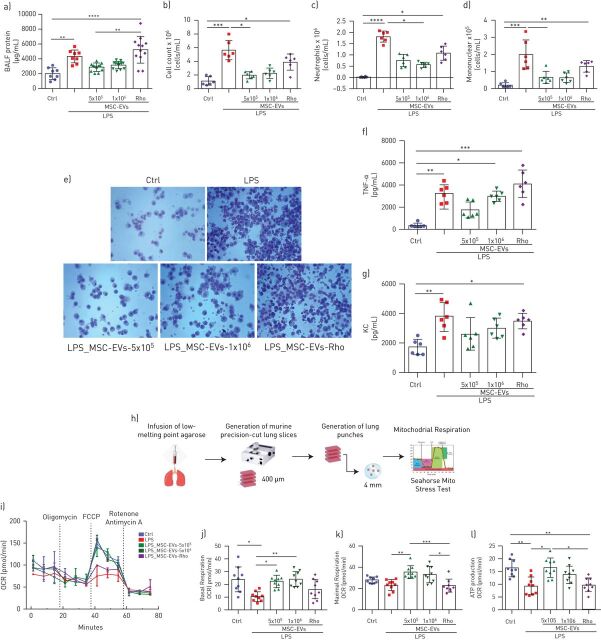
MSC-EVs reduce LPS-induced lung injury and restore mitochondrial respiration in the murine lung tissue *in vivo*. a) Total protein concentrations in the bronchoalveolar lavage fluid (BALF) samples (n=8–10 mice per group). b–d) Total cells counts (b), absolute neutrophil counts (c) and absolute mononuclear cell counts (d) in the BALF samples (n=6 mice per group). e) Representative images of BALF cytospin preparations demonstrating inflammatory cell recruitment to the airspaces were taken using the Leica Epifluorescence DM5500 microscope (original magnification, x20). f) BALF levels of tumour necrosis factor alpha (TNF-α). g) BALF levels of keratinocyte-derived chemokine (KC, murine analogue of interleukin-8). h) Schematic representation of generation of murine precision cut-lung slices (mPCLSs). i) Representative Seahorse Mito Stress test assay showing oxygen consumption rate (OCR) in mPCLSs. j–l) Calculated values for basal respiration (j), maximal respiration (k) and ATP production (l) of mPCLSs. Data represented as mean ±sd. *: p<0.05; **: p<0.01; ***: p<0.001; ****: p<0.0001. Kruskal-Wallis test with *post hoc* Dunn's test.

Given the above results, we hypothesised that extracellular vesicle-mediated mitochondrial transfer would improve mitochondrial dysfunction in an *in vivo* lung injury model. Effects of two different extracellular vesicle doses were compared: extracellular vesicles generated from 5×10^5^ and 1×10^6^ MSCs. Administration of either dose of control extracellular vesicles comparably decreased the LPS-stimulated increases in BALF total protein, total and differential cell counts, (figure 8a–d). Administration of Rho-EVs (generated from 1×10^6^ MSCs) had no effect. Cytospin preparations of BALF demonstrated substantial inflammatory cell recruitment to the alveolar compartment, consisting predominantly of neutrophils, in the LPS-injured group which was reduced by administration of extracellular vesicles but not Rho-EVs (figure 8e).

Extracellular vesicle but not Rho-EV treatment demonstrated a trend towards reduction of BALF levels of representative inflammatory cytokines (TNF-α and keratinocyte-derived chemokine) however the effect did not reach statistical significance (figure 8f and g).

To assess mitochondrial function, we measured mitochondrial respiration in the mouse lung tissue in this model using PCLSs (figure 8h). LPS-induced injury resulted in significant downregulation of basal and maximal mitochondrial respiration and of mitochondrial ATP turnover. Systemic administration of both doses of control extracellular vesicles but not Rho-EVs restored both mitochondrial respiration and ATP production (figure 8i–l).

## Discussion

The major findings from this study are the following. 1) MSC-EVs contain mitochondria, which are readily internalised by recipient cells and intercalate into the endogenous mitochondrial network (figures 1 and 2). 2) LPS-induced inflammatory response and increase in the epithelial and endothelial barrier permeability coupled with significant mitochondrial dysfunction. The transfer of mitochondria in MSC-EVs was crucial not only for the alleviation of mitochondrial dysfunction but also for the restoration of barrier integrity in both cells types, suggesting important role of mitochondria in the maintenance of alveolar-capillary barrier function (figures 2 and 3). 3) In both cell types, MSC-EVs were able to restore normal levels of mitophagy and mitochondrial biogenesis, which were perturbed by LPS (figure 4). 4) MSC-EVs mitochondrial transfer was able to attenuate mitochondrial dysfunction and restore mitochondrial respiration inhibited by LPS at the lung tissue level in PCLSs (figure 5). 5) Stimulation with ARDS plasma significantly impaired barrier integrity and mitochondrial function in both epithelial and endothelial cells, however therapeutic effect of MSC-EVs was only seen in the presence of plasma from patients with hypo-inflammatory phenotype (figures 6 and 7). 6) Therapeutic effect of MSC-EVs in the *in vivo* model of LPS-induced lung injury was dependent on the presence of mitochondria and EV mitochondrial transfer improved mitochondrial respiration in the lung tissue *in vivo* (figure 8).

Once questioned on the reality of their existence, MSC extracellular vesicles are now entering clinical arena as a potent alternative to the whole-cell therapy [16, 37, 38]. Islam *et al.* [18] was the first to report that MSC-EVs could act as vehicles to transfer functional mitochondria and restore bioenergetics of the host cells, compromised by inflammatory microenvironment. This was followed by a seminal publication by Phinney *et al.* [19], demonstrating that MSCs actively secrete mitochondria into extracellular vesicles, as a result of unfinished mitophagy. We have previously reported that mitochondrial transfer is important for MSC modulation of macrophages leading to their metabolic reprogramming towards anti-inflammatory phenotype and also promote wound healing capacity of human small airway epithelial cells [20, 21, 39]. Notably, recent publication reports that monocytes also are capable of mitochondrial secretion in the extracellular vesicles and that mitochondrial stress contributes to the inflammatory properties of their secretome [40]. Here we provide characterisation of the mitochondria present in the MSC-EVs by a range of techniques (figure 1). We demonstrate presence of vesicles larger than 400 nm which are not accountable for apoptotic bodies and possessing ultrastructural characteristics of mitochondria, presence of vesicles double-positive for MSC mitochondria and MSC-EV surface markers CD44 and CD63, and high level of expression of mitochondrial outer membrane protein in the extracellular vesicles (figure 1a–d). Importantly, we demonstrate that MSCs in which mitochondria were rendered dysfunctional by pre-treatment with Rhodamine-6G [20] also secrete extracellular vesicles and their concentration and size distribution profiles are comparable with extracellular vesicles secreted by normal MSC, except for the absence of vesicles above 400 nm in size, corresponding to the mitochondria (figure 1e).

Accumulating evidence indicate that alterations in the mitochondrial function play a role in development of many forms of critical illness [13, 41, 42]. However, the data on contribution of mitochondrial dysfunction to the disruption of the alveolar–capillary barrier in ARDS are limited. Islam *et al.* [18] were the first to consider that MSC therapeutic effect in lung injury is mediated through restoration of mitochondrial bioenergetics and ATP production of alveolar epithelial cells. A recent publication from the same group determined mechanistic links between mitochondrial dysfunction and microvascular hyperpermeability *in vivo* [43]. Here we demonstrate that LPS stimulation results in significant impairment of barrier integrity of primary human small airway epithelial and microvascular endothelial cells which is coupled with profound mitochondrial dysfunction and disturbance of mitochondrial biogenesis and mitophagy (figures 3 and 4). Donation of the exogenous mitochondria results in the restoration of normal mitochondrial respiration in both cell types to the normal levels and is critical for the therapeutic effect of extracellular vesicles on recovery of the epithelial and endothelial barrier functions.

In line with these findings, we observe that LPS stimulation impairs mitochondrial membrane potential, increases mtROS production and inhibits mitochondrial respiration in the human PCLSs, and mitochondrial transfer from MSC-EVs is crucial to alleviate mitochondrial dysfunction and restore mitochondrial respiration in this model (figure 5).

Overproduction of mtROS is thought to be crucial factor in the development of mitochondrial dysfunction leading to disruption of mitochondrial membrane integrity, dissipation of ΔΨ and further damage to the cell [44–46]. Antioxidant-based therapies are explored for clinical efficacy in multiple disease conditions including critical illness [47–49]. Interestingly, treatment with mitochondrial ROS inhibitor, MitoTempo, was sufficient to reverse inflammatory response but was not able to restore mitochondrial respiration (figures 5 and 6), suggesting that MSC-EVs effect cannot be fully explained by inhibition of mtROS production. It is plausible to hypothesise that upregulation of mitochondrial biogenesis observed with extracellular vesicle mitochondrial transfer could be important for alleviation of LPS-induced mitochondrial dysfunction.

Two biological subphenotypes of ARDS (hypo- and hyper-inflammatory) are retrospectively identified in four large clinical trials, including HARP-2 [7]. Interestingly, a subsequent study using microarray analysis of whole-blood gene expression in an observational cohort of 210 patients with sepsis-related ARDS found that one-third of genes were differentially expressed between phenotypes and patients in the “reactive” or “hyper-inflamed” group were characterised by expression of genes indicative of mitochondrial dysfunction [50]. We found that stimulation of epithelial as well as endothelial cells with ARDS plasma induces inhibition of mitochondrial respiration regardless of the phenotype (figure 6). MSC-EVs however were able to partially restore both mitochondrial function and barrier integrity only in hypo-inflammatory plasma and presence of mitochondria was central to this effect (figure 6), suggesting, that patients with hypo-inflammatory phenotype may be more responsive to the MSC-EVs treatment. It is plausible that in the setting of hyper-inflammatory microenvironment there is a threshold to endothelial and epithelial cell injury, beyond which retrieval of physiological bioenergetic function is hindered.

Ang-2 has been established as a key mediator and biomarker of endothelial injury in ARDS [31–35] and was the single biomarker which levels were significantly reduced after MSC administration in the START study [36]. It is important to highlight that in the present study control but not Rho-EVs demonstrated strong trend towards reduction of Ang-2 secretion levels by both HPMECS and SAECS in the presence of LPS and ARDS plasma, indicating relevance of extracellular vesicle mitochondrial transfer for alleviation of the severity of endothelial injury (figure 7).

To confirm the importance of MSC-EVs mitochondrial transfer in the lung injury *in vivo*, endotoxin-injured mice were given extracellular vesicles derived from normal or Rhodamine-6G-treated MSCs. Normal extracellular vesicle, but not Rho-EVs, were able to significantly reduce the extent of lung injury after 24 h (figure 8). These data are in line with several previously published reports demonstrating therapeutic effects of MSC-EVs in the *in vivo* models of ARDS [26, 51–55]. Furthermore, measurements of oxygen consumption rates of the lung tissue in this model using mPCLSs, demonstrated that instillation of endotoxin significantly inhibited mitochondrial respiration and ATP production in the mouse lungs and extracellular vesicle mitochondrial transfer was able to restore mitochondrial respiration to normal levels (figure 8).

This study has limitations. We used ARDS plasma diluted to 10% by volume; while exposing cells to all constituents of the ARDS microenvironment, this effectively reduced the concentrations of the stimuli. We acknowledge that BALF would be more suitable environment to stimulate epithelial cells, unfortunately BALF samples from both phenotypes were not available. The endotoxin-induced lung injury model is relatively mild and does not reflect complexity of human ARDS; the primary aim of the *in vivo* experiments was to provide a proof of principle that MSC-EVs are capable to alleviate mitochondrial dysfunction *in vivo*. Additionally, we did not investigate molecular mechanisms by which mitochondria in extracellular vesicles are protective in lung injury and specifically molecular mechanisms responsible for alleviation of endothelial barrier dysfunction, this is the subject of ongoing work. We did not use fibroblasts-derived extracellular vesicles as cell specific control for *in vivo* experiments, however previous studies have demonstrated that fibroblast-derived extracellular vesicles had no therapeutic effects in LPS and *Escherichia coli*-induced murine lung injury models [55, 56]. Our primary focus in this study was to investigate the mechanistic role of MSC-EV-mediated mitochondrial transfer, therefore we used Rho-EVs which contain similar profile of vesicles but lack mitochondria, we believe this would constitute a better control.

In conclusion, we for the first time, have demonstrated that ARDS environment induces significant mitochondrial dysfunction in human lung tissue implicated in the impairment of alveolar–capillary barrier functions. Therapeutic effect of MSC-EVs on the restoration of barrier integrity is mediated by mitochondrial transfer.

## Supplementary material

10.1183/13993003.02978-2020.Supp1 **Please note:** supplementary material is not edited by the Editorial Office, and is uploaded as it has been supplied by the author.
Supplementary methods ERJ-02978-2020.Supplement
Supplementary figure S1 ERJ-02978-2020.Figure_S1
Supplementary video S1 ERJ-02978-2020.Video_S1
Supplementary video S2 ERJ-02978-2020.Video_S2

## Shareable PDF

10.1183/13993003.02978-2020.Shareable1 This one-page PDF can be shared freely online.
Shareable PDF ERJ-02978-2020.Shareable
