# Supplementary material for: Mesenchymal stromal cell extracellular vesicles rescue mitochondrial dysfunction and improve barrier integrity in clinically relevant models of ARDS
Source: Eur Respir J. 2021 Jul 1;58(1):2002978. doi: 10.1183/13993003.02978-2020 (PMC8318599; doi:10.1183/13993003.02978-2020)
Supplement: Supplementary file 1 [file ERJ-02978-2020.Supplement.pdf]

Supplementary Materials and Methods

Bone Marrow-derived Mesenchymal Stromal Cells (MSCs)

Human bone marrow-derived Mesenchymal Stromal Cells (MSCs) were obtained from American Type Culture Collection (ATCC® PCS-500-012). These cells had been extensively characterised in accordance with the standard laid out by the International Society for Cellular Therapy (ISCT) [1]. MSCs were cultivated in a T175 flask (ThermoFisher Scientific, UK) with 15 mL alpha-Minimum Essential Medium (α-MEM) supplemented with 16.5% heat-inactivated foetal calf serum (FCS), 1% L-glutamine amino acid (4mM) and 1% Penicillin/Streptomycin (50ug/mL) (all from Gibco, ThermoFisher Scientific, UK) and maintained at 37° C in a humidified tissue culture incubator at 5% CO_2 until used for experimentation. MSCs were fed every 2-3 days and sub-cultured into new flasks whenever 70-80% confluency was achieved. Upon reaching 80% confluence, media was aspirated from the flask of cells, washed with Phosphate Buffered Saline (DPBS) to remove any FCS-containing medium and MSCs were passaged with 0.05% trypsin-EDTA solution (Gibco, ThermoFisher Scientific, UK) for up to 5 minutes at 37° C until the cells had detached from the culture surface. Trypsin activity was neutralised by addition of α-MEM complete medium at a volume equal to that of the trypsin added to the flask, cells collected and centrifuged for 5 minutes at 1200 rpm. Following centrifugation, the supernatant was aspirated off and the cell pellet was resuspended in α-MEM complete medium. Cell viability, density and final concentration were then determined by Trypan Blue exclusion and by counting in a hemocytometer (Neubauer model). MSCs were used at passage 5. Multiple MSC donors were used for experiments through this study.

Human Primary Lung Small Airway Epithelial Cells (HSAECs) and Human Pulmonary Microvascular Endothelial Cells (HPMECs)

Human Primary Lung Small Airway Epithelial Cells (HSAECs) were obtained from PromoCell (PromoCell, Germany) in proliferating passage 2 and used to passage 8. These cells are isolated from the distal portion of the human respiratory tract in the 1 mm bronchiole area. They stain positive for cytokeratin. Cells were cultivated in small airway epithelial growth medium supplemented mix medium (PromoCell, Germany) (Table S1) and 1% Penicillin/Streptomycin (50ug/mL) (Gibco, ThermoFisher Scientific, UK). Human Pulmonary Microvascular Endothelial Cells (HPMECs) were obtained from PromoCell (PromoCell,

Germany) in proliferating passage 2 and used to passage 8. These cells were isolated from the lung blood and lymphatic capillaries microvascular endothelial cells. Cells were characterized by their expression of Cluster of Differentiation 31 (CD31) and Von Willebrand factor (VWF) and lack of expression of smooth muscle alpha-actin. HPMECs were cultivated in endothelial cell growth medium MV supplemented mix medium (PromoCell, Germany) (Table S1) and 1% Penicillin/Streptomycin (50ug/mL) (Gibco, ThermoFisher Scientific, UK).

HSAECs and HPMECs were cultivated in a T25 flask (ThermoFisher Scientific, UK) and maintained at 37° C in a humidified tissue culture incubator at 5% CO_2 until used for experimentation. Both cells type were passaged when they reached approximately 80% confluency. DetachKit (PromoCell, Germany) reagents were used at room temperature (RT). Media was aspirated from the culture flask, washed with 30mM HEPES Buffered Saline solution (HEPES-BSS; PromoCell, Germany), aspirated and the cells incubated with Trypsin-EDTA (0.04%/0.03%) solution for approximately 5 minutes at room temperature until the cells had detached from the culture surface. Trypsin was neutralised by adding a volume of Trypsin Neutralisation Solution (NTS; PromoCell, Germany) equal to the volume of trypsin. Detached cells were collected, centrifuged at 220g for 3 minutes. Following centrifugation, the supernatant was aspirated off and cell pellet resuspended in appropriated cell culture medium. Cell viability, density and final concentration were then determined by Trypan Blue exclusion and by counting in a hemocytometer (Neubauer model). Cells counted were seeded for experiments or culture flasks for expansion.

Table S1: Final concentrations of supplements after addition to basal medium

**Small Airway Epithelial Cell Growth Medium**	**Endothelial Cell Growth MV Medium**
0.004 mL/mL Bovine Pituitary Extract	0.05 mL/mL Fetal Calf Serum
10 ng/mL Epidermal Growth Factor (recombinant human)	0.004 mL/mL Endothelial Cell Growth Supplement
5 ug/mL Insulin (recombinant human)	10 ng/mL Epidermal Growth Factor (recombinant human)
0.5 ug/mL Hydrocortisone	90 ug/mL Heparin
0.5 ug/mL Epinephrine	1 ug/mL Hydrocortisone
6.7 ng/mL Triiodo-L-thyronine	
10 ug/mL Transferrin (recombinant human)	
0.1 ng/mL Retinoic Acid	
2.5 mg/mL Bovine Serum Albumin-Fatty Acid Free (BSA-FAF)	

Mesenchymal Stromal Cells-derived extracellular vesicles (MSC-EVs) isolation

The MSC-EVs ultracentrifugation was adapted from Zhu et al [2]. MSCs were grown to ~ 80% confluence in T175 flask under standard conditions. To prevent potential contamination with serum EVs, MSCs were cultured in 10 mL serum-free α-MEM supplemented medium for 48 hours to generate EVs. Conditioned medium (MSC-CM) was collected in 50 mL falcon tubes and centrifuged at 4° C for 2000 g for 45 minutes to remove cellular debris. The supernatant was transferred into polycarbonate ultracentrifuge tube (Beckman Coulter, US) ensuring that they were equally balanced. The tubes were attached to a 70 Ti rotor in Beckman Coulter ultracentrifuge (Optima Max-XP Ultracentrifuge, Beckman-Coulter, US) and centrifuged at 4° C for 100.000g for a further 3 hours to isolate MCS-EVs (acceleration =9 and deceleration = 5). The supernatant was gently aspirated, MSC-EVs gently washed with PBS and resuspended in PBS according to the final MSC cell count (EVs generated from 10^6 MSCs correspond to 10 µl of PBS).

MSC-EVs protein and RNA quantification

Quantification of MSC-EVs preparations was based on previously published work and findings in our group [3]. After MSC-EVs isolation, the concentration of RNA was assessed using the Nanodrop 2000 (ThermoFisher Scientific, UK) and protein assessed by BCA protein assay (Micro BCA protein assay kit, ThermoFisher Scientific, UK). 10 µl of MSC-EVs generated from $1x10^6$ MSC were used for *in vitro* experiments for each $1x10^4$ primary cells (HSAECs and HPMECs).

MSC-EVs measurements with Nanosight NS300

Size distribution and concentration of MSC-EVs were performed using Nanoparticle tracking analysis (NTA) device NanoSight NS300 (Malvern, UK). After MSC-EVs isolation from $1x10^6$ MSCs cell density, all samples were diluted in distilled water to a final volume of 1 mL. Settings were set according to the manufacturer’s software manual (NanoSight NS300 User Manual). The detection threshold was determined to include as many particle as possible with the restrictions that 10-100 red crosses were counted while < 10% were not associated with distinct particles. Autofocus was adjusted so that indistinct particles were avoided. For each measurement, five 1-min videos were captured at 25° C. After capture, videos have been analysed by the in-build NanoSight Software NTA.

Visualisation of MSC-EVs morphology using transmission electron microscopy (TEM)

After MSC-EVs isolation, 500 µl of 4% paraformaldehyde (PFA) was added very slowly to the MSC-EVs pellet and incubated at RT for 30 minutes. After that, PFA was aspirated and washed three times with PBS for 10 minutes each. The MSC-EVs were resuspended in 30 µl of PBS and formvar/carbon-coated grids (Science Services, München) were loaded with 10 µl of sample. The grids and samples were incubated for 20 minutes at RT, dried on filter paper (Whatman, UK) and fixed with 2% glutaraldehyde for 10 minutes. Using non-magnetc tweezers, the grids were gently moved across five drops of distilled water for 1 minute each and dried between washes. After that, a drop of 1% tannic acid ACS reagent (Sigma Aldrich, UK) was added for 40 minutes at RT, washed two times in PBS for 1 minutes each, dried and then placed on a drop of TAAB EM heavy metal stain 336 (TAAB, Laboratory Equipment LTD, UK) for 30 minutes at RT. The grids were quickly submerged in 50% ethanol, washed in distilled water and dried. MSC-EVs were visualised on a TEM microscope (JEOL, JEM 1400Plus, Japan).

Characterization of MSC-EVs by Flow cytometry

For characterization by Flow cytometry, flow cytometry was realized with the addition of aldehyde/sulphate latex beads (ThermoFisher Scientific, UK) with 4um of diameter [4]. For mitochondrial identification, MitoTracker Deep Red FM (ThermoFisher Scientific, UK) was used for labelling mitochondria in MSCs respectively. MSC were grown in T175 to 80% confluence, washed with PBS to remove any residual FCS before staining and pre-stained with 200 nM of MitoTracker Deep Red FM in 10 mL serum-free α-MEM and incubated in 1x104 primary cells (HSAECs and HPMECs) 1x104 primary cells (HSAECs and HPMECs) the dark for 45 minutes. The dye-containing medium was aspirated, and the cells washed five times with PBS, 10 mL serum-free α-MEM supplemented medium was added for 48 hours to generate EVs as previously described. The latex beads were used to provide a reference point to compare with the EVs in FSC vs SSC plots. 100 µl of each fraction isolated were incubated with 0.25 µl of aldehyde/sulfate-latex beads (ø = 4 µm; 5.5×10^6 particles/ml) for 20 minutes at RT. Then 1 mL of PBS supplemented with 0.1% BSA (Roche) and 0.01% NaN_3 (G-Biosciences) was added and the sample incubated overnight on rotation. Bead-coupled EVs were centrifugated at 2000 g for 10 minutes, wash with 1 mL of BCB and centrifuged again. The pellet was resuspended with 100 µl of PBS and stained with anti-CD63 (Biolegend,) FITC-conjugated, anti-CD44 (eBioscience) PE-Cy7 conjugated primary antibodies for 45 minutes at 4° C. Incubation of the beads-coupled EVs with respective isotype control Ab was used as

negative controls. After incubation, MSC-EVs were washed twice and re-suspended in PBS. Gating of EV-decorated 4 µm in diameter beads was performed based on FCS/SSC parameters, so then unbound EVs or possible antibody aggregates were excluded from the analysis. Analysis was performed in FACS CantoII flow cytometer in FACSDiva software and data analysis was performed using FlowJo software (FlowJo, Ashland, OR).

Western blot analysis of MSC-EVs lysates

MSC-EVs obtained from healthy MSCs or MSCs with dysfunctional mitochondria (MSC-EVs-Rho) were lysed using Radioimmunoprecipitation assay (RIPA) buffer supplemented with cOmplete EDTA-free protease inhibitor (Sigma Aldrich) and phosphatase inhibitor PhosSTOP (Sigma Aldrich). The MSC-EVs were resuspended in 50 µl of RIPA buffer, incubated at 4° C for 30 minutes and placed in an ice-cold sonication for 1 minute. After that, gently mixed on ice for 2 minutes. Protein concentration was determined using BCA protein assay (Micro BCA protein assay kit, ThermoFisher Scientific, UK). 40 µg of total protein of each sample was separated on 10% SDS-PAGE under reducing conditions and transferred onto a PVDF membrane (GE Healthcare, Germany). The membranes were blocked with 5% milk and incubated with rabbit anti-human primary antibody anti-TOM20 (1:1000, Cell Signaling) overnight at 4° C. After washing, the blots were incubated with HRP-conjugated anti-rabbit IgG secondary antibody (1:1000, Cell Signaling) for 2h at RT. Protein expression was visualised using SuperSignal™ West Femto Maximum Sensitivity Substrate (ThermoFisher Scientific, UK) at G:Box Chemi imaging system (Syngene).

Generation of MSC-EVs dysfunctional mitochondria (MSC-EVs-Rho)

Loss-of-function experiments were performed to assess the importance of mitochondrial component of extracellular vesicles. To produce dysfunctional mitochondria, MSCs were treated with Rhodamine-6G (Sigma Aldrich) at a concentration of 1 µg/mL for 48 hours at 37° C. Once mitochondrial respiration would be blocked, MSCs had their medium supplemented to support glycolysis. Standard α-MEM was further supplemented with uridine (Sigma Aldrich) to produce a concentration of 50 ug/mL and sodium pyruvate to a concentration of 2.5 mM (Sigma Aldrich). After 48 hours, MSCs were washed five times with PBS and CM was generated for EVs isolation as previously described. MSC-EVs-Rho were resuspended in PBS according to the final MSC cell count (EVs generated from 10^6 MSCs correspond to 10

µl of PBS). Size and concentration of MSC-EVs-Rho was quantified using NanoSight NS300 as previously described.

In vitro stimulation experiments

To generate an inflammatory microenvironment, *E. coli* lipopolysaccharide (LPS) 0111:B4 (Merck) was added to the HSAECs and HPMECs at working concentration of 1 µg/mL in 1% complete medium for 24 hours. In other experiments, plasma samples from ARDS patients recruited to the HARP-2 clinical trial were used for stimulation. These samples have been previously classified into 2 phenotypes based on concentration of inflammatory biomarkers [5] . Plasma samples from 20 patients (10 from each phenotype) were pooled to generate a stock and the pooled sample was then diluted in 1% complete medium to final concentration of 10% before stimulation. Plasma samples from healthy volunteers were used as control.

Studying mitochondrial transfer through MSC-EVs

For mitochondrial transfer study, MitoTracker fluorescent probes (ThermoFisher Scientific, UK) were used to label active mitochondria in MSCs. MSC were grown in T175 to 80% confluence, washed with PBS to remove any residual FCS before staining and pre-stained with 200 nM of MitoTracker Deep Red FM (ThermoFisher Scientific, UK) into 10 mL serum-free α-MEM and incubated in darkened conditions for 45 minutes. The dye-containing medium was aspirated, the cells were washed five times with PBS and 10 mL serum-free α-MEM supplemented medium was added for 48 hours to generate EVs as previously described. The last wash was stored and added to control wells to confirm that all residual staining was removed during wash steps. HSAECs and HPMECs at density of $1x10^4$ cells/well were plated in 96-well black plate with clear bottom (Nunc, ThermoFisher Scientific, UK) and incubated in normal cell cultured conditions to allow cell adhere to the plate. HPMECs or HSAECs were washed with HEPES-BSS and stained with 200 nM of MitoTracker Green (ThermoFisher Scientific, UK) in the dark for 45 minutes to stain endogenous mitochondria. After that, cells were washed and stimulated with *E.coli* LPS and treated with PBS, last wash collected from MSCs or MSC-EVs isolated from MSCs pre-stained with MitoTracker Deep Red FM for 24 hours to allow EVs uptake. Cells were washed three times and 100 µl of PBS was added. The images were taken using a Nikon 6D Eclipse Ti-E inverted microscope with Okolab touch temperature unit and CO_2 environmental chamber (Nikon Instruments, Japan) (40x dry super plan fluor ELWD objective with 0.6 NA). 3D reconstruction of multiple z-stacks,

deconvolution and video of microscopy images were done using Nikon 6D Eclipse software. The degree of colocalization of fluorescence signal originating from endogenous mitochondrial (MitoTracker Green) and MSC-EVs mitochondrial transfer (MitoTracker Deep Red FM) was quantified by calculating Manders' distinct colocalization coefficient M1 using Nikon 6D Eclipse software.

Epithelial and Endothelial permeability to FITC-Dextran

HSAECs and HPMECs were seeded at a density of $1x10^4$ cells/cm2 on transwell inserts with 0.4 µm pores suitable for a 24 well plate (Greiner). Cells were cultured at 37° C in a humidified tissue culture incubator at 5% CO_2 with medium changes every 2-3 days until confluent monolayer formation. Medium leak from the upper chamber into the lower chamber was analysed to confirm the confluence and ensure the formation of a good barrier. Following monolayer formation, cells were stimulated with *E.coli* LPS and treated with 10 µl of PBS, MSC-EVs or MSC-EVs-Rho for 24 hours. After that, medium was collected and stored for further analysis, 100 µl of 1% medium with 10 µl of 70 kDa FITC-Dextran (Sigma Aldrich) was added to the upper chamber and 500 µl of 1% medium to the lower chamber. A positive control in which FITC-Dextran was added to inserts on which no cells had been seeded was included. A blank in which PBS was added in place of FITC-Dextran to the upper chamber of inserts containing no cells was also included. Cells were kept in tissue culture incubator for 30 minutes, 100 µl samples from the lower chamber were collected and added to a 96-well black plate with clear bottom (Corning) in triplicate. Fluorescence intensity was read immediately using a FLUOstar Omega microplate reader at 485nm excitation and 520nm emission. Results are presented as % of positive control.

Cell impedance measurements in xCELLigence RTCA Single-Plate (SP) System

The xCELLigence RTCA SP system (Agilent) was used to measure, process and analyse the impedance (Cell index), which was detected by sensor electrodes in E-plate 16 (ACEA Biosciences). Before addition of cells, E-plate was coated with 2% gelatin (Sigma Aldrich), washed three times with PBS to remove the excess of gelatin and leave in the tissue culture hood to dry. The background was measured before seeding the cells in the E-plate, placed at 37° C in a humidified incubator at 5% CO_2 with 50 µl of 1% complete media. Then, 100 µl with $2x10^4$ HSAECs or HPMECs were seeded with 1% complete media and the plate was kept at RT for 1 hour to allow cells to adhere. After that, E-plate was set into RTCA SP Station (ACEA Biosciences) in the CO_2 incubator and connected to the RTCA analyser. The baseline

impedance was measured to ensure that all wells and connections were working within acceptable limits. As cells adhere to the E-plates, the Cell Index value increases from zero to a a maximum cell index of ~ 8 corresponding to very strongly adherent monolayer. Once cells measurements reach a plateau, the medium was changed and cells stimulated with *E.coli* LPS or plasma samples, treated with 20 µl of PBS, MSC-EVs or MSC-EVs-Rho for 24 hours. In separate experiments, before *E.coli* LPS or ARDS plasma samples stimulation, cells were pre-treated with 5 µM of MitoTempo (Mt), a mitochondria-targeted superoxide dismutase antioxidant, 4 hours prior stimulation. The cell index was recorded every 15 min for 24 hour. Every independent experiment was performed in triplicate. To demonstrate the effect of treatments, the cell index was normalized to equal value of cell index before adding the stimulations to making the values more comparable. The stimulated medium was collected and stored for further analysis.

Measurements of mitochondrial respiration

XFp96 analyzer from Seahorse Bioscience (Agilent) was used to determine cells' oxygen consumption rate (OCR) following manufacturer's instructions. HSAECs and HPMECs ($1x10^4$ cells/well) were plated 2 days before the assay into XFp cell culture mini-plates. Three replicate wells (technical triplicates) were used for each condition. During this incubation, a cartridge plate provided with the Cell Mito Stress Test Kit (Agilent Technologies) was soaked in XF calibrant solution and left to incubate at 37º C and 0% CO_2. 24 hours before measurements, cells were stimulated with *E.coli* LPS or plasma samples from ARDS endotypes and treated with 10 µl of PBS, MSC-EVs or MSC-EVs-Rho for 24 hours. In separated experiments, before *E.coli* LPS or ARDS plasma samples stimulation, cells were pre-treated with 5 µM of MitoTempo (Mt) 4 hours prior stimulation. After that, supernatant was collected and stored for further analysis and cells washed with HEPES-BSS. 180 µl of XF assay medium was added to each well at final concentration of 10 mM of Glucose, 1mM of Pyruvate and 2 mM of L-glutamine (all from Agilent), and placed in a humidified tissue culture incubator to incubate at 37º C and 0% CO_2 for 1 hour before experimentation. The mitochondrial stress test was performed using the following final concentrations of inhibitors: 1.0 µM oligomycin, 1.0 µM of carbonyl cyanide p-trifluoromethoxyphenylhydrazone (FCCP) and 0.5 µM of Rotenone/Antimycin-A (Rot/AA) (all from Agilent). After each inhibitor was added to the medium, three sequential measurements were taken. Each well was normalized to the percent of initial OCR. The mitochondrial stress test parameters in mitochondrial stress test medium

were calculated using the Wave software (Version 2.2) (Agilent Technologies) according to the manufacturer's instructions.

Determination of cell viability by Lactate Dehydrogenase (LDH) assay

LDH was quantified using Cytotoxicity Detection Kit (Roche) following manufactures instructions. Cell supernatants were centrifuged to remove cell debris at 5000 rpm for 10 minutes at 4º C. Supernatant was collected and 50 µl samples were added in triplicate into a 96-well plate (Nunc, ThermoFisher Scientific, UK). 50 µl of reaction mixture was added to the samples, mixed on a plate shaker for 1 minute and incubated for 30 minutes in the dark at RT. A positive control was included where cells were lysed with a final concentration of 2% Triton-X (Sigma Aldrich) 10 minutes before collection of supernatants. Optical densities were measured at 405nm using a FLUOstar Omega microplate reader. Data were analysed using MARS data analysis software. Results are presented as % relative to the positive control.

Enzyme-linked Immunosorbent Assay (ELISA)

Human TNF-α, IL-8, advanced glycosylation end-product specific receptor (RAGE) and human angiopoietin-2 (ANG-2) in cell and PCLS supernatants and murine TNF-α and keratinocyte chemoattractant (KC-murine IL-8 homolog) in broncho-alveolar lavage fluid were quantified using ELISA duoset kits (R&D System, Biotechne) according to manufacturer instructions. The ELISA plates were analyzed using Versamax spectrophotometer set to read at 450nm and 540nm wavelength. A subtraction of the wavelength absorbance readings at 540nm was taken from the readings at 450nm for correction. 4-parameter standard curves were produced using Softmax Pro v2.6 and concentrations of samples were extrapolated.

Investigation of mitochondrial biogenesis

5-bromo-2-deoxyuridine (BrdU, Sigma Aldrich) was used to assess the production of newly synthetised MtDNA within cells [6]. HSAECs and HPMECs were seeded in µ-slide 8-well chambers at density of $2x10^4$ cells per well and allowed to form a monolayer. A 10mM stock concentration of BrdU was diluted to a working concentration of 10 µM in 1% complete medium and added to the cells, *E. coli* LPS and treatment with 20 µl PBS, MSC-EVs or MSC-EVs-Rho was added for 24 hours. After that, medium was collected and stored, cells washed with HEPES-BSS and fixed with 4% PFA for 15 minutes. After fixation, cells were washed and permeabilised with 0.1% Triton-X-100 (Sigma Aldrich) in 1x PBS for 15 minutes at RT.

HSAECs and HPMECs DNA was denatured by adding 200 µl of 2N hydrochloric acid for 30 minutes at 37° C. To amplify the BrdU signal, a highly sensitive AlexaFluor 488 Tyramide SuperBoost Kit (ThermoFisher Scientific, UK) was used. Endogenous peroxidases were blocked with 100 µl 3% hydrogen peroxide solution for 30 minutes. After that, cells were washed and incubated with 100 µl 10% normal goat serum (NGS) supplied in the kit for 1 hour at RT to block non-specific antibody binding. A 1:50 dilution of purified monoclonal anti-BrdU antibody (0.5 mg/mL; Biolegend, UK) and the same concentration of IgG isotype control diluted in 1% NGS with 0.1% Tween 20 (Sigma Aldrich) were added to the wells and incubated overnight at 4° C. The wash step was repeated, cells incubated with 150 µl poly-HRP-conjugated secondary antibody supplied in the kit for 1 hour at RT. The wash step was repeated and 100 µl of tyramide solution was added to the cells and incubated for approximately 10 minutes at room temperature in the dark. The reaction was then terminated with a 1:11 dilution of stop reagent supplied in the kit and cells washed. The chamber was removed and coverslips were mounted on glass slides with Prolong Gold containing DAPI (ThermoFisher Scientific, UK). Fluorescence signals were viewed on the Leica SP8 confocal microscope with a 40× oil-immersion objective. Analysis of BrdU-labeled mtDNA was performed using Image J software. The cytosolic volume of each cell was measured by subtracting the volume of the nucleus from the total cell volume. All experiments were performed on cultured cells from at least three separate occasions with at least two replicates per condition.

Assessment of the mitophagy levels

The protocol for this experiment was adapted from Widdrington et al [7]. HSAECs and HPMECs were seeded in µ-slide 8-well chambers at density of $2x10^4$ cells per well and allowed to form a monolayer. Cells were stimulated with *E. coli* LPS and treated with 20 µl of PBS, MSC-EVs or MSC-EVs-Rho for 24 hours. Serum starved cells were used as a positive control for the induction of mitophagy and treatment with 5nM bafilomycin A1 (Sigma Aldrich) for 2h was used to prevent autophagosome turnover. 24 hours after cell stimulation, medium was collected and stored, cells were washed with HEPES-BSS and stained with 200 nM of MitoTracker Deep Red FM for 45 minutes at 37° C in the dark. After that, cells were washed and fixed with ice-cold 100% methanol at -20° C for 20 minutes. After fixation, cells were incubated with 100 µl 5% FCS with 0.3% Triton X-100 in PBS for 1 hour at RT to block non-specific antibody binding. Cells were incubated with LC3-II primary antibody FITC conjugated (1:100; Cell Signaling) in 1% BSA with 0.3% Triton X-100 in 1x PBS overnight at 4° C in the dark. The wash step was repeated, cells counterstained with 300nM DAPI solution

for 3 minutes at RT in the dark. The wash step was repeated, chamber was removed, and coverslips were mounted on glass slides with Prolong Gold Antifade Reagent (ThermoFisher Scientific, UK). Co-localisation of LC3A/B and endogenous mitochondria stained with MitoTracker Deep Red FM was determined using the Leica SP8 confocal microscope with a 40× oil-immersion objective. Co-localisation was assessed using the Mander's M1 co-localisation coefficient using Leica LAS X software.

Measurement of mitochondrial membrane potential

5,5',6,6'-tetrachloro-1,1',3,3'-tetraethylbenzimidazolylcarbocyanine iodide (JC-1) dye was used to assess mitochondrial membrane potential. This dye accumulates within mitochondria contingent on mitochondrial membrane potential. Red/green JC-1 fluorescence ratio is proportional to mitochondrial membrane potential. HSAECs and HPMECs were seeded at density of $1x10^4$ in 96-well black plate with clear bottom (Nunc) in triplicate and incubated in normal cell culture conditions to allow cell adhere to the plate. After stimulation, cells were washed and stained with JC-1 (Abcam) at a concentration of 20 µM diluted in 1x dilution buffer supplied in the kit for 15 minutes at 37° C in the dark. A control was included where 100 µM of FCCP was simultaneously added to the cells at the time of JC-1 staining to induce mitochondrial depolarisation. Following staining, wells were washed three times using 1x dilution buffer. 100 µl of PBS was added per well and live cells were immediately imaged at 20x magnification, using the EVOS FL Auto epifluorescent microscope. Red and green fluorescence intensity of each image was measured in ImageJ and the red/green ratio was calculated.

Detection of mitochondrial superoxide production in live cells

MitoSOX Red (ThermoFisher Scientific, UK) fluorescent dye allowing for a selective detection of superoxide in the mitochondria of live cells was used to detect mitochondrial superoxide production. HSAECs and HPMECs were seeded at density of $1x10^4$ in 96-well black plate with clear bottom (Nunc, ThermoFisher Scientific, UK) in triplicate and incubated in normal cell culture conditions to allow cell to adhere to the plate. Cells were stimulated with *E. coli* LPS or plasma samples from ARDS endotypes and treated with 10 µl of PBS, MSC-EVs, MSC-EVs-Rho or pre-treated with 5 µM of MitoTempo (Mt), a mitochondria-targeted superoxide dismutase antioxidant, 4 hours prior stimulation. 24 hours after cell stimulation, supernatants were collected and stored, cells washed with HEPES-BSS and MitoSOX was added at 5 µM in the medium and incubated for 20 minutes at 37° C in the dark. Following

staining, cells were washed three times, counterstained with 20 µM Hoechst (ThermoFisher Scientific, UK; for a final of 10 µM) and 100 µl of PBS was added per well. Live cells were immediately imaged at 20x magnification, using the EVOS FL Auto epifluorescent microscope. Red fluorescence intensity of each image was measured using ImageJ.

Generation of human Precision-Cut Lung Slices (PCLSs)

Lungs obtained from organ donors (where the organ has been unsuitable for transplantation, and next of kin has consented for use in research) were used for lung slices cultured *ex vivo*. Ethical approval has been given by National Review Ethics Service (NRES) in association with NHS Blood and Transplant for lungs obtained within the UK (REC 14/LO/0250). PCLSs were prepared from lung tissue donated by three donors. The protocol for PCLSs preparation was adapted from Franziska E. Uhl et al [8]. Lung segments were cannulated via a visible bronchus and filled with warm low gelling temperature agarose (2% by weight, Sigma, kept at 40°C) diluted in sterile cultivation medium (DMEM/Ham's F12; Gibco, supplemented with 100 U.mL-1 penicillin, 100 µg.mL-1 streptomycin, Sigma). Lungs segments were cooled on ice to allow gelling of the agarose. Cylindrical cores were separated using tissue coring tool, followed by cutting using a vibratome (Compresstome VF-300; Precisionary Instruments) to create 400 µm-thick slices. Incubation medium was changed every 30 min for 2 h after slicing, in order to remove residual agarose and cell debris from the tissue. The PCLSs were cultured in medium supplemented with 0.1% fetal calf serum (FCS) (Gibco) in 96 wells plates at 37° C in humidified incubator containing 5% CO_2 under submerged conditions with daily changes of medium. PCLSs were punched to a diameter of 4 mm using a biopsy punch, ensuring exclusion of major airways. Punches were taken from similar peripheral areas of the lung to minimise sample variation and standardise for tissue volume in further analysis.

***In vitro* stimulation of human PCLSs**

To generate an inflammatory microenvironment, *E. coli* LPS 0111:B4 (Merck) was added to the PCLSs at working concentration of 1 µg/mL in 1% complete medium for 24 hours. PCLSs were treated with 10 µl of PBS, MSC-EVs, MSC-EVs-Rho (isolated from 10^6 MSCs) or pre-treated with 5 µM of MitoTempo (Mt), a mitochondria-targeted superoxide dismutase antioxidant, 4 hours prior stimulation. 24 hours after stimulation measurements was performed, supernatants were collected and stored for further analysed.

Viability of human PCLSs

Viability of tissue slices or 4 mm biopsy punches was checked by Lactate Dehydrogenase (LDH) assay using Cytotoxicity Detection Kit (Roche) as previously described. Results are presented as % relative to the positive control.

Measurement of mitochondrial respiration in human PCLSs

For measurement of mitochondrial respiration, 4 mm biopsy punches were cultured in the XF 96-well tissue culture plates. The Seahorse XF Cell Mito Stress Test Kit (Agilent Technologies) was used as previously described. The mitochondrial stress test was performed using the following final concentrations of inhibitors: 1.0 µM oligomycin, 1.0 µM of carbonyl cyanide p-trifluoromethoxyphenylhydrazone (FCCP) and 0.5 µM of Rotenone/Antimycin-A (Rot/AA) (all from Agilent). Oxygen consumption rate (OCR) readouts produced by the assay were analysed using the Wave software (Version 2.2) (Agilent Technologies).

Detection of mitochondrial membrane potential and mitochondrial superoxide production in human PCLSs

4 mm biopsy punches were used for measurements of mitochondrial membrane potential using JC-1staining and mitochondrial superoxide production using MitoSOX Red staining as described above. Images were taken using a Nikon 6D Eclipse Ti-E inverted microscope with and Okolab touch temperature unit and CO_2 environmental chamber (Nikon Instruments, Japan) (20x dry objective). The analysis was performed using ImageJ software.

Studying mitochondrial transfer through MSC-EVs in PCLSs

For mitochondrial transfer study, MitoTracker fluorescent probes (ThermoFisher Scientific, UK) were used to label active mitochondria in MSCs as previous described. PCLSs stimulated with E.coli LPS and treated with last wash collected from MSCs (PBS), MSC-EVs isolated from MSCs pre-stained with MitoTracker Deep Red FM or MSC-EVs-Rho for 24 hours to allow EVs uptake. PCLSs were washed three times with PBS, removed, and coverslips were mounted on glass slides with Prolong Gold Antifade Reagent (ThermoFisher Scientific, UK). The images were taken using a Leica SP8-Upgright Confocal microscopy (original magnification x20 HC PL FLUOTAR, scale bar 50µm).

***In vivo* studies**

C57BL/6 mice (4 to 6 weeks old, Harland Institute, UK) were used. Animals were maintained in the Biological Service Unit (BSU) at Queen’s University Belfast. Animals were housed in

standard laboratory cages, with access to food and water *ad libitum*. Experiments were sanctioned and approved by the UK Home Office and Queen's University Belfast Ethical Review Committee.

Animal preparation and experimental protocol

Mice were anaesthetized with were anesthetized with 100 µL of xylazine/ketamine (0.25 $mg.kg^{-1}$ and 0.025 $mg.kg^{-1}$, respectively) intraperitoneally. Lung injury was induced by administering *E. coli* LPS O111:B4 (Merck) intratracheally (2mg/kg body weight) diluted in PBS at a volume of 50 µl. In control mice, sterile PBS was administered intratracheally (50 µl) instead. Four hours later, mice were then treated with vehicle control (PBS), MSC-EVs (isolated from $5x10^5$ or $1x10^6$ MSC cells, 50 µl diluted in PBS) or MSC-EVs-Rho ($1x10^6$, 50 µl diluted in PBS). Mice were observed until recovery and returned to holding. 24 hours after LPS instillation, mice were culled and BALF was collected for cell counts, protein quantification and cytokine analysis.

Bronchoalveolar lavage fluid

Mice were culled with intraperitoneal injection of xylazine/ketamine. Thereafter, the trachea was cannulated by 21-gauge needle and 1mL of PBS was instilled into the lungs. The PBS was gently flushed in and lifted out 3-5 times to ensure thorough sampling of the bronchoalveolar compartment was collected. BALF samples were centrifuged at 5000 g for 5 mins to remove the cells from suspension. Supernatants were collected and stored for future processing.

Bronchoalveolar Lavage Fluid (BALF) Cellularity and Total Protein Content

The cell pellet was resuspended in 300 µl of PBS and first diluted 2-fold. 20 µl of the diluted sample was mixed with 20 µl of trypan blue and then 10 µl was added to Neubauer chamber and total leukocyte counting was performed under light microscopy. BALF sample and dilutions were accounted for the final counts expressed as cells/mL. After taking the aliquot for total cell counts, the cell pellet was centrifuged at 10,000 rpm for 5 mins using the StatSpin Cytofuge®2 (Beckman Coulter, VWR) onto microscope slides. Slides were given 2 hours to dry and then stained using the Speedy Diff kit (Clin-tech). Differential cell count was performed in cytospin smears using the Leica Epifluorescence DM5500 microscope at 20x magnification. Enough images were taken of each slide to count a total of 400 cells. Cells were counted using ImageJ software and the percentage of neutrophils was determined. The total

protein content in the BALF supernatant was quantified by BCA protein assay (Micro BCA protein assay kit, ThermoFisher Scientific, UK).

Generation of murine PCLSC (mPCLs)

24 hours after LPS instillation, mice were culled with a mixture of xylazine /ketamine. The trachea was sectioned, cannulated with severed 18-gauge needle, the anterior chest wall was surgically removed, and the lungs exposed. Using a syringe, lungs were filled *in situ* with warm, low gelling temperature agarose (3% by weight, Sigma, kept at 40C) diluted in sterile cultivation medium (DMEM/Ham's F12; Gibco, supplemented with 100 U.mL-1 penicillin, 100 µg.mL-1 streptomycin, Sigma). The trachea was ligated with thread to retain the agarose inside the lung. The lung was excised, transferred into a 50 mL conical tube with cultivation medium and cooled on ice for 15 minutes to allow gelling of the agarose. The lobes were separated and cut with a vibratome to a thickness of 400 µM. A total of 12 to 15 slices were made per lung. After slicing, biopsies punches using a 4mm disposable biopsy punch were performed. The mPCLs were cultured in medium supplemented with 0.1% fetal calf serum (FCS) (Gibco) in 96 wells plates. Incubation medium was changed every 30 min for 2 h after slicing, to remove agarose residues and cell debris from the tissue.

Assessing mitochondrial respiration on mPCLSs

To evaluate mitochondrial respiration, the Seahorse XF Cell Mito Stress Test Kit (Agilent Technologies) was used as previously described. mPCLs punches were transferred into XF 96-well tissue culture plates. mPCLSs were washed with XF basal medium and 180 µl of XF basal medium was added to each well. Oligomycin, carbonyl cyanide-4 (trifluoromethoxy) phenylhydrazone (FCCP) and rotenone/antimycin-A inhibitors were prepared as per the manufacturer's instructions. Oxygen consumption rate (OCR) readouts produced by the assay were analysed using the Wave software (Version 2.2) (Agilent Technologies).

Statistical Analysis

Analysis was performed using GraphPad Prism 6 software (GraphPad Software, La Jolla, CA). Experiments were performed at least in triplicate, the average of three technical replicates was taken as a single data point for each donor, and the points were pooled together for statistical analysis. The Kolmogorov-Smirnov test with Lilliefors' correction was used to evaluate the homogeneity of variances. For parametric data, one-way analysis followed by post-hoc analysis using Bonferronis' was selected. For nonparametric data, the Kruskal-Wallis test followed by

post-hoc using Dunns' comparisons was used. The statistical significance level was set as $p < 0.05$.

References

1. Dominici, M., et al., *Minimal criteria for defining multipotent mesenchymal stromal cells. The International Society for Cellular Therapy position statement.* Cytotherapy, 2006. **8**(4): p. 315-7.
2. Zhu, Y.G., et al., *Human mesenchymal stem cell microvesicles for treatment of Escherichia coli endotoxin-induced acute lung injury in mice.* Stem Cells, 2014. **32**(1): p. 116-25.
3. Morrison, T.J., et al., *Mesenchymal Stromal Cells Modulate Macrophages in Clinically Relevant Lung Injury Models by Extracellular Vesicle Mitochondrial Transfer.* Am J Respir Crit Care Med, 2017. **196**(10): p. 1275-1286.
4. Suarez, H., et al., *A bead-assisted flow cytometry method for the semi-quantitative analysis of Extracellular Vesicles.* Sci Rep, 2017. **7**(1): p. 11271.
5. Calfee, C.S., et al., *Acute respiratory distress syndrome subphenotypes and differential response to simvastatin: secondary analysis of a randomised controlled trial.* Lancet Respir Med, 2018. **6**(9): p. 691-698.
6. Lentz, S.I., et al., *Mitochondrial DNA (mtDNA) biogenesis: visualization and duel incorporation of BrdU and EdU into newly synthesized mtDNA in vitro.* J Histochem Cytochem, 2010. **58**(2): p. 207-18.
7. Widdrington, J.D., et al., *Exposure of Monocytic Cells to Lipopolysaccharide Induces Coordinated Endotoxin Tolerance, Mitochondrial Biogenesis, Mitophagy, and Antioxidant Defenses.* Front Immunol, 2018. **9**: p. 2217.
8. Uhl, F.E., et al., *Preclinical validation and imaging of Wnt-induced repair in human 3D lung tissue cultures.* Eur Respir J, 2015. **46**(4): p. 1150-66.
