# Supplementary material for: Mesenchymal stromal cell extracellular vesicles rescue mitochondrial dysfunction and improve barrier integrity in clinically relevant models of ARDS
Source: Eur Respir J. 2021 Jul 1;58(1):2002978. doi: 10.1183/13993003.02978-2020 (PMC8318599; doi:10.1183/13993003.02978-2020)
Supplement: Supplementary file 2 [file ERJ-02978-2020.Figure_S1.pdf]

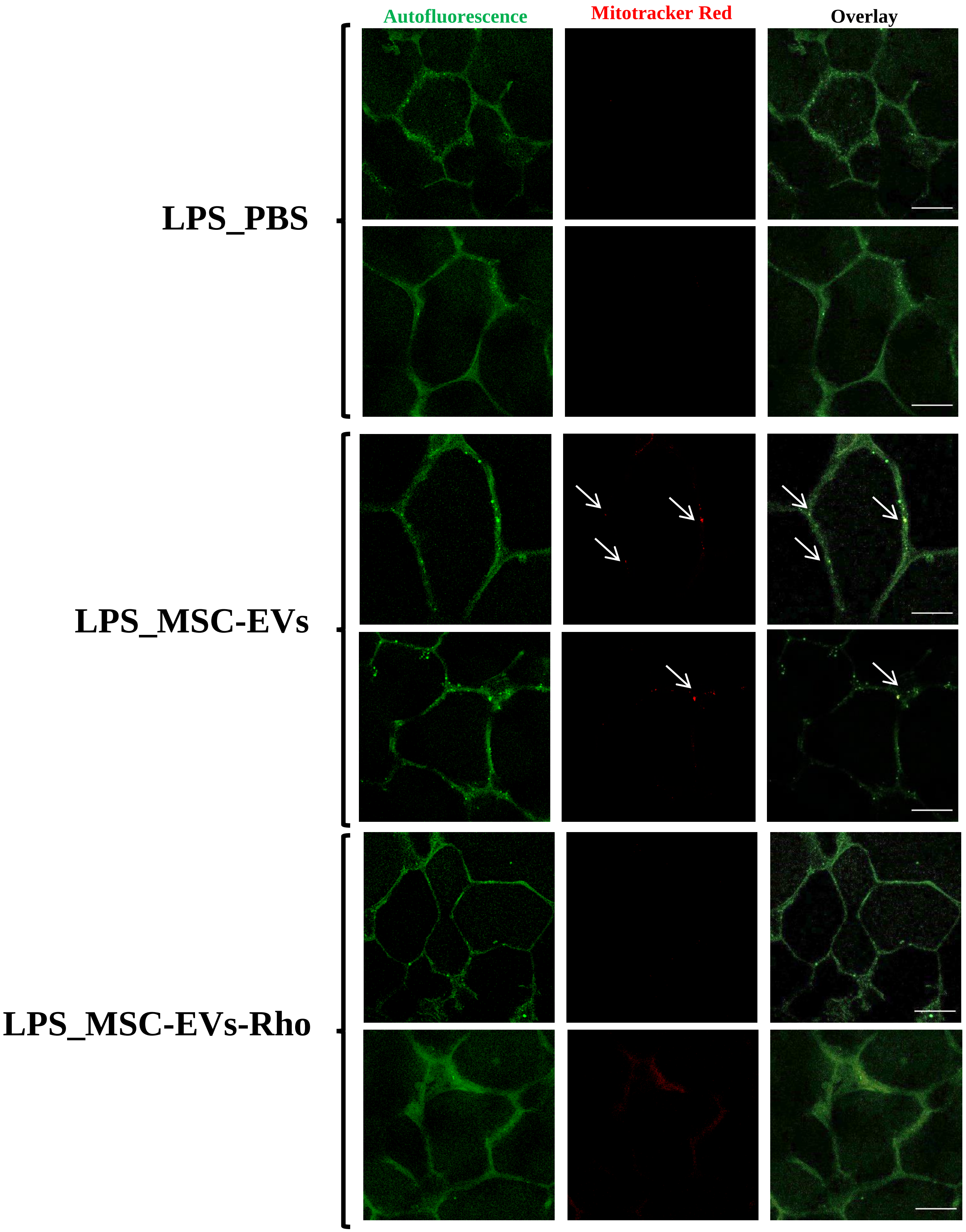

Supplementary Figure 1. Mitochondrial transfer through MSC-EVs to Human Precision Cut Lung Slices (PCLSs). Representative confocal images of MSC-EVs mitochondria transfer in PCLSs. PCLSs were stimulated with LPS and treated with PBS, MSCs pre-stained with MitoTracker Deep Red FM or MSC-EVs-Rho for 24 hours to allow EVs uptake. The images were taken using the Leica SP8-Upgright Confocal microscopy (scale bars 50 µm).
