# Supplementary material for: Mesenchymal stromal cell extracellular vesicles rescue mitochondrial dysfunction and improve barrier integrity in clinically relevant models of ARDS
Source: Eur Respir J. 2021 Jul 1;58(1):2002978. doi: 10.1183/13993003.02978-2020 (PMC8318599; doi:10.1183/13993003.02978-2020)
Supplement: Supplementary file 5 [file ERJ-02978-2020.Shareable.pdf]

EUROPEAN RESPIRATORY JOURNAL
ORIGINAL RESEARCH ARTICLE
J. DUTRA SILVA ET AL.

SHAREABLE PDF

Mesenchymal stromal cell extracellular vesicles rescue mitochondrial dysfunction and improve barrier integrity in clinically relevant models of ARDS

Johnatas Dutra Silva [1], Yue Su[1], Carolyn S. Calfee[2,3,4], Kevin L. Delucchi[5], Daniel Weiss[6], Danny F. McAuley [1], Cecilia O'Kane[1] and Anna D. Krasnodembskaya [1]

[1]Wellcome-Wolfson Institute for Experimental Medicine, School of Medicine, Dentistry, and Biomedical Sciences, Queen's University Belfast, Belfast, UK. [2]Dept of Medicine, Division of Pulmonary, Critical Care, Allergy and Sleep Medicine, University of California, San Francisco, San Francisco, CA, USA. [3]Dept of Anesthesia, University of California, San Francisco, San Francisco, CA, USA. [4]Cardiovascular Research Institute, University of California, San Francisco, San Francisco, CA, USA. [5]Dept of Psychiatry, University of California, San Francisco, San Francisco, CA, USA. [6]Dept of Medicine, Larner College of Medicine, University of Vermont, Burlington, VT, USA.

Corresponding author: Anna D. Krasnodembskaya (a.krasnodembskaya@qub.ac.uk)

Check for updates

Shareable abstract (@ERSpublications)
This study demonstrates that mitochondrial dysfunction is an important mechanism of ARDS pathogenesis. Mitochondrial transfer is crucial for the ability of MSC extracellular vesicles to restore integrity of the alveolar–capillary barrier. https://bit.ly/2JuqoCY

Cite this article as: Dutra Silva J, Su Y, Calfee CS, *et al*. Mesenchymal stromal cell extracellular vesicles rescue mitochondrial dysfunction and improve barrier integrity in clinically relevant models of ARDS. *Eur Respir J* 2021; 58: 2002978 [DOI: 10.1183/13993003.02978-2020].

This single-page version can be shared freely online.

Copyright ©ERS 2021.

This version is distributed under the terms of the Creative Commons Attribution Licence 4.0.

This article has supplementary material available from erj.ersjournals.com

Received: 3 Aug 2020
Accepted: 2 Dec 2020

Abstract

Alveolar epithelial–capillary barrier disruption is a hallmark of acute respiratory distress syndrome (ARDS). Contribution of mitochondrial dysfunction to the compromised alveolar-capillary barrier in ARDS remains unclear. Mesenchymal stromal cells-derived extracellular vesicles (MSC-EVs) are considered as a cell-free therapy for ARDS. Mitochondrial transfer was shown to be important for the therapeutic effects of MSCs and MSC-EVs. Here we investigated the contribution of mitochondrial dysfunction to the injury of alveolar epithelial and endothelial barriers in ARDS and the ability of MSC-EVs to modulate alveolar–capillary barrier integrity through mitochondrial transfer.

Primary human small airway epithelial and pulmonary microvascular endothelial cells and human precision cut lung slices (PCLSs) were stimulated with endotoxin or plasma samples from patients with ARDS and treated with MSC-EVs, barrier properties and mitochondrial functions were evaluated. Lipopolysaccharide (LPS)-injured mice were treated with MSC-EVs and degree of lung injury and mitochondrial respiration of the lung tissue were assessed.

Inflammatory stimulation resulted in increased permeability coupled with pronounced mitochondrial dysfunction in both types of primary cells and PCLSs. Extracellular vesicles derived from normal MSCs restored barrier integrity and normal levels of oxidative phosphorylation while an extracellular vesicles preparation which did not contain mitochondria was not effective. *In vivo*, presence of mitochondria was critical for extracellular vesicles ability to reduce lung injury and restore mitochondrial respiration in the lung tissue.

In the ARDS environment, MSC-EVs improve alveolar–capillary barrier properties through restoration of mitochondrial functions at least partially *via* mitochondrial transfer.

cc BY
